# Phase 1 trial of the oral AKT inhibitor MK-2206 plus carboplatin/paclitaxel, docetaxel, or erlotinib in patients with advanced solid tumors

**DOI:** 10.1186/1756-8722-7-1

**Published:** 2014-01-03

**Authors:** L Rhoda Molife, Li Yan, Joanna Vitfell-Rasmussen, Adriane M Zernhelt, Daniel M Sullivan, Philippe A Cassier, Eric Chen, Andrea Biondo, Ernestina Tetteh, Lillian L Siu, Amita Patnaik, Kyriakos P Papadopoulos, Johann S de Bono, Anthony W Tolcher, Susan Minton

**Affiliations:** 1 Drug Development Unit, The Institute of Cancer Research/The Royal Marsden NHS Foundation Trust, Downs Road, Sutton, Surrey SM2 5PT, UK; 2 Merck & Co., Inc., One Merck Drive, Whitehouse Station, NJ 08889, USA; 3 H. Lee Moffitt Cancer Center & Research Institute, 12902 Magnolia Drive, Tampa, FL 33612, USA; 4 Drug Development Programme, Princess Margaret Hospital, 610 University Avenue, 5-700, Toronto, ON M5G 2 M9, Canada; 5 South Texas Accelerated Research Therapeutics (START), 4383 Medical Drive, San Antonio, TX 78229, USA; 6 Division of Clinical Sciences, The Institute of Cancer Research, 15 Cotswold Road, Belmont, Sutton, Surrey SM2 5NG, UK; 7 Department of Medicine, Centre Léon Bérard, 28 rue Laennec, 69008 Lyon, France

**Keywords:** MK-2206, AKT inhibitor, Protein serine-threonine kinase, Phase 1, Chemotherapy, Combination therapy, Solid tumors

## Abstract

**Background:**

Inhibition of AKT with MK-2206 has demonstrated synergism with anticancer agents. This phase 1 study assessed the MTD, DLTs, PK, and efficacy of MK-2206 in combination with cytotoxic and targeted therapies.

**Methods:**

Advanced solid tumor patients received oral MK-2206 45 or 60 mg (QOD) with either carboplatin (AUC 6.0) and paclitaxel 200 mg/m^2^ (arm 1), docetaxel 75 mg/m^2^ (arm 2), or erlotinib 100 or 150 mg daily (arm 3); alternative schedules of MK-2206 135-200 mg QW or 90-250 mg Q3W were also tested.

**Results:**

MTD of MK-2206 (N = 72) was 45 mg QOD or 200 mg Q3W (arm 1); MAD was 200 mg Q3W (arm 2) and 135 mg QW (arm 3). DLTs included skin rash (arms 1, 3), febrile neutropenia (QOD, arms 1, 2), tinnitus (Q3W, arm 2), and stomatitis (QOD, arm 3). Common drug-related toxicities included fatigue (68%), nausea (49%), and rash (47%). Two patients with squamous cell carcinoma of the head and neck (arm 1; Q3W) demonstrated a complete and partial response (PR); additional PRs were observed in patients (1 each) with melanoma, endometrial, neuroendocrine prostate, NSCLC, and cervical cancers. Six patients had stable disease ≥6 months.

**Conclusion:**

MK-2206 plus carboplatin and paclitaxel, docetaxel, or erlotinib was well-tolerated, with early evidence of antitumor activity.

**Trial registration:**

ClinicalTrials.gov: NCT00848718.

## Introduction

The phosphatidylinositide 3-kinase (PI3K)/AKT/mammalian target of rapamycin (mTOR) signaling pathway is a critical driver of tumor progression [1]. Hyperactivation of this pathway is an important driver of malignant progression via increased cancer cell growth, survival, and metabolism, as well as chemoresistance [2,3]. Hyperactivation may occur through different mechanisms, including upstream stimulation by receptor tyrosine kinases, PIK3CA and AKT mutations or amplifications, and loss of PTEN function [4]. In view of the key role of the PI3K/AKT/mTOR pathway in cancer, multiple strategies have been developed in recent years to target critical components of this signaling cascade [5-7]. AKT (protein kinase B [PKB]), a serine/threonine kinase, is directly activated in response to PI3K and is a major effector of PI3K in cancers [8-10]. There are 3 different protein isoforms, AKT1, AKT2, and AKT3, with overlapping and distinct roles in cancer; for example, AKT1 promotes cellular survival and growth [11]. In addition, AKT activation and overexpression are commonly associated with chemo- and radio-resistance [2,12], and dominant-negative mutants of AKT have been shown to enhance the activity of chemotherapeutics [13,14].

Adenosine triphosphate (ATP) competitive and allosteric classes of small-molecule AKT inhibitors with varying potencies and specificities for the different AKT isoforms have been developed [5,7]. One member of the allosteric class is MK-2206 (Merck & Co., Inc., Whitehouse Station, NJ, USA), an oral, highly selective inhibitor of AKT that binds at a site in the pleckstrin-homology (PH) domain, distinct from the ATP-binding pocket, resulting in a conformational change that prevents the localization of AKT to the plasma membrane and its subsequent activation [15,16]. It displays nanomolar (nM) potency against all 3 AKT isoforms (AKT1, half maximal inhibitory concentration [IC50] = 5 nM; AKT2, IC50 = 12 nM; AKT3, IC50 = 65 nM) [16]. A first-in-human phase 1 combination study defined the maximum tolerated dose (MTD), pharmacokinetics (PK), and pharmacodynamics (PD) of an alternate day (QOD) and weekly (QW) schedule of MK-2206 in patients with advanced solid tumors [17,18]. The dose-limiting toxicities (DLT) were rash and stomatitis. The PK profile was dose proportional, and PD analysis of both schedules demonstrated the downstream effects of AKT inhibition with a significant decline in phosphorylated AKT (pAKT; ser473) in post-treatment tumor biopsies, and in pPRAS40 (rhr246) in hair follicles. Reversible hyperglycemia and an increase in insulin c-peptide further confirmed target modulation. Minor responses were demonstrated in 2 patients with neuroendocrine pancreatic cancers and 1 patient with pancreatic adenocarcinoma and PTEN loss (in addition to KRAS G12D mutation).

In preclinical models, MK-2206 enhanced the activity of conventional cytotoxics and other molecularly targeted therapies [19]. In vitro, MK-2206 demonstrated synergy with both erlotinib and lapatinib in inhibiting proliferation and inducing apoptosis of non-small cell lung (NSCLC) cell lines, including those that were RAS mutant, and breast cancer cell lines. Although treatment with erlotinib inhibited EGFR and pERK phosphorylation of the RAF-RAS-MEK pathway in the A431 cell line and mouse NCI-H292 tumor xenografts, there was no effect on pAKT and pRAS40, downstream markers of AKT inhibition. However, the combination with MK-2206 resulted in decreased levels of pAKT and pRAS40. The inhibition of both pathways led to more profound inhibition of pGSK3b and pS6, which are downstream signaling proteins that correlate with cell growth and survival. The combination also demonstrated significantly greater in vivo tumor growth suppression and tumor regressions over each single agent using both a 3 times per week and QW schedule of MK-2206 in the mouse tumor xenografts.

In vitro, MK-2206 demonstrated synergy with several conventional cytotoxics, including carboplatin and docetaxel, in inhibiting the growth of NCI-H292 and A2780 tumor cells [19]. Carboplatin-induced apoptosis was also enhanced by MK-2206 in a sequence-dependent manner: concurrent treatment or pretreatment with carboplatin induced A2780 cell death in a dose-dependent manner, whereas pretreatment with MK-2206 did not. In vivo, MK-2206 synergised with docetaxel, carboplatin, and gemcitabine in inhibiting the growth of PC-3 prostate and NCI-H462 tumor xenografts with a similar-sequence dependent pattern as for carboplatin in vitro.

On this background, a multi-arm phase 1 dose-escalation study of MK-2206 in combination with carboplatin and paclitaxel, docetaxel, or erlotinib in patients with advanced solid tumors was initiated. The primary objectives were to evaluate safety and tolerability, DLTs, and the MTD/recommended phase 2 dose (RP2D) of MK-2206 when administered orally (PO) in the above combinations. Additional objectives were to explore the PK profile, antitumor activity of MK-2206 in combination and correlation of anti-tumor activity with tumor P13K pathway activation events.

## Materials and methods

This phase 1, multi-arm, open-label, dose-escalation study (ClinicalTrials.gov: NCT00848718; http://clinicaltrials.gov/ct2/show/NCT00848718) was conducted at 4 centers (Royal Marsden NHS Foundation Trust, Sutton, Surrey, UK; South Texas Accelerated Research Therapeutics [START], Texas, USA; Princess Margaret Hospital, Toronto, Ontario, Canada; H. Lee Moffitt Cancer Center and Research Institute, Tampa, Florida, USA). The study was conducted in accordance with the Declaration of Helsinki and Good Clinical Practice Guidelines of the International Conference on Harmonization and was approved by the Ethics Committees and Institutional Review Boards at all study sites. All patients provided written informed consent before any study procedures were performed.

### Eligibility criteria

Patients 18 years or older with confirmed advanced solid tumors were eligible if they had progressed after standard therapy, or if no standard therapy was available for them; had Eastern Cooperative Oncology Group performance status ≤1; surgery or chemotherapy within the previous 4 weeks; ≤3 prior lines of cytotoxic therapies (arms 1 and 2 only); residual toxicity from prior treatment grade ≤1; adequate bone marrow, renal, and hepatic function; and fasting serum glucose ≤1.1× the upper limit of normal and hemoglobin A1c (HbA1c) ≤8%. Patients were excluded if they were diabetic and on antidiabetic therapy, pregnant or breastfeeding, receiving oral corticosteroids, had any condition(s) that would impede drug ingestion or absorption, or had other significant coexisting medical conditions.

### Study design

MK-2206 was initially administered PO every other day (QOD) on days 1, 3, 5, and 7 (days 1-7) of a 21-day cycle, in combination with intravenous (IV) carboplatin (area under the curve 6.0 mg/mL [AUC 6]) over 1 hour and IV paclitaxel 200 mg/m^2^ over 3 hours (arm 1; Table 1); or IV docetaxel 75 mg/m^2^ over 1 hour (arm 2; Table 1). MK-2206 was also administered QOD continuous with daily PO erlotinib 100 mg or 150 mg every 21 days; both MK-2206 and erlotinib were given on a 21-day cycle (arm 3; Table 1). Based on the MTD of single-agent MK-2206 of 60 mg QOD, determined using a modified 3 + 3 design [20], cohorts of 3 to 6 patients were to be treated at preplanned MK-2206 dose levels of 45 mg and 60 mg, in combination with carboplatin and paclitaxel (arm 1) or docetaxel (arm 2), or with erlotinib (arm 3).

**Table 1:** MK-2206 treatment regimen by treatment arm

**Treatment arm**	**MK-2206 dose, mg**	**Schedule**^**a**^	**Combination with:**
1	45	QOD	Carboplatin (IV, AUC6, 1-hour infusion) and paclitaxel (IV, 200 mg/m^2^, 3-hour infusion)
	60		
	90	Q3W	
	135		
	200		
2	45	QOD	Docetaxel (IV, 75 mg/m^2^, 1-hour infusion)
	90	Q3W	Docetaxel (IV, 60 mg/m^2^, 1-hour infusion)
	135		
	200		
3	45	QOD*	Erlotinib (oral, 100 mg, QD)
	45		Erlotinib (oral, 150 mg, QD)
	135	QW	Erlotinib (oral, 100 mg, QD)
	135		Erlotinib (oral, 150 mg, QD)

*Abbreviations*: *IV* intravenous, *AUC6* area under the curve 6.0 mg/mL, *QD* once daily.

^a^QOD = once every other day on days 1, 3, 5, and 7 of 21-day cycle, except *: alternate day dosing on days 1–21; Q3W = once every 3 weeks on day 1 of 21-day cycle; QW = once weekly on days 1, 8, and 15 of 21-day cycle.

During dose escalation of the days 1–7 QOD dosing schedule of MK-2206, emerging data led to the introduction of 2 protocol amendments. First, data from the same schedule in the first-in-human phase 1 study demonstrated that MK-2206 had a long half-life (t_1/2_) of 60 to 80 hours. The tolerability of a QW schedule was investigated and found to be acceptable with evidence of PD activity [17]. Preclinical efficacy studies had also demonstrated the antitumor effect of MK-2206 administered either QW or 3 times per week with daily erlotinib [19]. This suggested that continuous exposure with MK-2206 may not be necessary with erlotinib and that overall, more flexible dosing schedules can be used in combinations [18]. Second, 3 DLTs of febrile neutropenia were reported at the first dose level of 45 mg MK-2206 QOD with IV docetaxel at 75 mg/m^2^. Consequently, 2 schedules (QW and Q3W) for MK-2206 were added to the current study (Table 1). Fasted patients received MK-2206 as 5-mg, 25-mg, or 200-mg tablets with chemotherapy or erlotinib. The dose-escalation phase in all schedules followed a toxicity probability interval approach, where the aim was to target a dose with a DLT rate of 30% [20]. Patients could continue receiving single-agent MK-2206 after completing chemotherapy or erlotinib doses.

### Safety

For all treatment schedules, safety assessments were conducted at baseline and on days 1, 2, 3, 7, 15, and 21 of cycle 1, and weekly in cycles 2 to 6. From cycle 7 onwards, safety assessments were performed on day 1 of each cycle. All patients had a history, physical examination including full ophthalmologic assessment, electrocardiogram, hematology and chemistry profiling, and urine analysis performed at baseline. In addition to glucose monitoring, serum c-peptide and whole blood HbA1c were measured at baseline and monthly. Adverse events (AEs) and laboratory variables were assessed using the National Cancer Institute Common Terminology Criteria for Adverse Events (NCI-CTCAE) version 3.0 ^1^.

A DLT was defined as any of the following occurring during the first cycle of treatment: grade 4 neutropenia lasting ≥7 days; grade 3 or 4 neutropenia with fever ≥38.5°C and/or infection requiring therapy; grade 4 thrombocytopenia; any drug-related AE that led to dose modification of MK-2206 or erlotinib; unresolved drug-related toxicity regardless of grade that resulted in a 3-week or longer delay of the start of cycle 2; persistent increase in QTc interval (>60 ms from baseline and/or >500 ms); clinically significant bradycardia; and any grade 3–5 nonhematologic toxicity with the exception of, in the opinion of the investigator, grade 3 nausea, vomiting, diarrhea, dehydration or hyperglycemia in the setting of inadequate compliance with supportive care treatment, alopecia, inadequately treated hypersensitivity reaction, and grade 3 elevated transaminases lasting 1 week or less.

### Pharmacokinetic analyses

In arms 1 and 2, for days 1–7 QOD dosing, blood sampling for MK-2206 PK was performed in cycle 1 on day 1 (predose, 2, 4, 6, 10, and 24 hours postdose), day 3 (48 hours postdose), day 7 (predose and 4 hours postdose), and days 15 and 21 (same time as day 1 predose sampling). For the Q3W schedule, samples were taken in cycle 1 on days 1 to 3 as per the QOD schedule, then on days 5, 7, 15, and in cycle 2 on day 1. Blood samples were collected predose and just before the end of the infusion for carboplatin, paclitaxel, and docetaxel for archival and possible PK analysis. Another sample was taken 30 minutes into the infusion of paclitaxel. These samples were archived for possible future analysis to investigate if any unexpected toxicities may have been as a result of a PK interaction. Docetaxel PK samples were analyzed in view of the DLT of febrile neutropenia observed in arm 2; however, PK parameters, such as half-life (t_1/2_) or systemic exposure of paclitaxel, docetaxel, carboplatin, and erlotinib, could not be evaluated due to the sparse blood sampling design used in this study.

In arm 3, for MK-2206 PK analysis in the QOD schedule, sampling was performed in cycle 1 on days 1 and 3 as in arms 1 and 2, then on days 7 and 15 (predose), day 21 (predose, 2, 4, 6, and 10 hours postdose), and in cycle 2 on days 1 and 2 (predose). For QW dosing, samples were taken in cycle 1 on day 1 (predose, 2, 4, 6, 10, and 24 hours postdose), day 3 (48 hours postdose), and day 5 (96 hours postdose), then on days 8 and 15 (predose). This sampling schedule was repeated in cycle 2, except for the day 15 predose sample, which was omitted. For the QOD schedule, PK sampling for erlotinib was performed in cycle 1 on day 1 (predose, 2 and 4 hours postdose) and day 21 (2 and 4 hours postdose); for the QW schedule, sampling was performed in cycle 1 on day 1 (predose, 2 and 4 hours postdose) and in cycle 2 on day 1 (2 and 4 hours postdose). Blood samples for MK-2206 PK were obtained, processed, and analyzed as described [18]. Blood samples were not analyzed for erlotinib concentrations as the potential for a marked drug-drug interaction with erlotinib as a victim was considered to be low as erlotinib is metabolized by both CYP3A and CYP1A. In addition, the CYP3A induction or inhibition potential of MK-2206 is low at the clinical concentrations achieved in this study, although the effect of MK-2206 on CYP1A is unknown.

### Biomarker studies

Circulating nucleic acids were analyzed for *PIK3CA* (exons 9 and 20), *KRAS* (exons 2 and 3)*,* and *BRAF* (exons 11 and 15) mutations. DNA extracted from whole blood samples was subjected to real-time quantitative polymerase chain reaction and spectrophotometric analysis as part of the quality control process, as previously described [21]. Mutation screening was performed using Surveyor Nuclease (Transgenomic, Inc., Nebraska, USA).

### Tumor response

Radiologic assessment (computed tomography [CT] and/or magnetic resonance imaging [MRI] scans) of disease status was performed at baseline and every 6 weeks according to Response Evaluation Criteria in Solid Tumors (v1.0) [22]. Relevant markers were used to assess the effects of MK-2206 combination therapy on various tumor types.

## Results

Seventy-two patients were treated between April 2009 and May 2012 (Table 2)—however, full accrual to the protocol was suspended due to a change in the developmental plans of MK-2206. In arm 1, 31 patients received a median of 3 cycles (range 1–10) of combination therapy; 10 of these patients went on to receive single-agent MK-2206 and completed a median of 4 cycles of treatment (range 1–15). In arm 2, 16 patients received a median of 3 cycles (range 1–7) with 1 of these patients receiving 1 further cycle of MK-2206 only. Another 25 patients received a median of 5 cycles (range 1–12) in arm 3 of the study.

**Table 2:** Patient characteristics

**Characteristic**	**Patients**
	**(N = 72)**
Age, years	
Median (SD)	58.0 (12.4)
Range	23–78
Gender, n (%)	
Male	36 (50.0)
Female	36 (50.0)
ECOG PS, n (%)	
0	22 (31)
1	49 (68)
Not recorded	1 (1)
Tumor type, n (%)	
NSCLC	13 (18.0)
Breast	10 (13.8)
Melanoma	6 (8.3)
Pancreas	5 (6.9)
Prostate	4 (5.6)
Colon	4 (5.6)
Esophagus	3 (4.2)
Parotid	3 (4.2)
SCLC	3 (4.2)
Other^a^	21 (29.2)

*Abbreviations*: *SD* standard deviation, *ECOG* Eastern Cooperative Oncology Group, *PS* performance status, *SCLC* small cell lung cancer, *SCC* squamous cell cancer.

^a^Cervical, cholangiocarcinoma, gastric, mesothelioma, endothelial cell, bladder, renal, sarcoma, SCC orbit, SCC vagina, SCC vulva, anal, tonsil, hard palate, and vulva.

### Dose-limiting toxicities and maximum tolerated dose

The MTD for all of the combinations could not be determined according to the predefined protocol criteria based on the number of patients enrolled at the time of study discontinuation—in cases where higher dose levels were not explored a maximum administered dose (MAD) was determined. Dose-escalation levels and DLTs are summarized in Table 3.

**Table 3:** Dose schedules and dose-limiting toxicities of MK-2206 in combination therapy

**Treatment arm**		**MK-2206 dosing schedule**	**Evaluable patients**	**Dose-limiting toxicities**
1	Carboplatin AUC 6	45 mg QOD^b^	5	1; rash
		60 mg QOD^b^	4	3^a^; rash, febrile neutropenia (2)
		90 mg Q3W	5	1; rash
	Paclitaxel 200 mg/m^2^			
		135 mg Q3W	5	1; TCP
		200 mg Q3W	5	2; rash
2	Docetaxel 75 mg/m^2^	45 mg QOD^b^	5	3; febrile neutropenia
	Docetaxel 60 mg/m^2^	90 mg Q3W	3	0
		135 mg Q3W	3	0
		200 mg Q3W	4	1; tinnitus
3	Erlotinib 100 mg	45 mg QOD^c^	8	2; rash, stomatitis
		135 mg QW	4	0
	Erlotinib 150 mg	45 mg QOD^c^	3	1; rash
		135 mg QW	5	1; rash

*Abbreviation*: *TCP* thrombocytopenia.

^a^3 events in 2 patients.

^b^days 1–7.

^c^continuous.

### QOD dosing schedule

**Figure 1:**
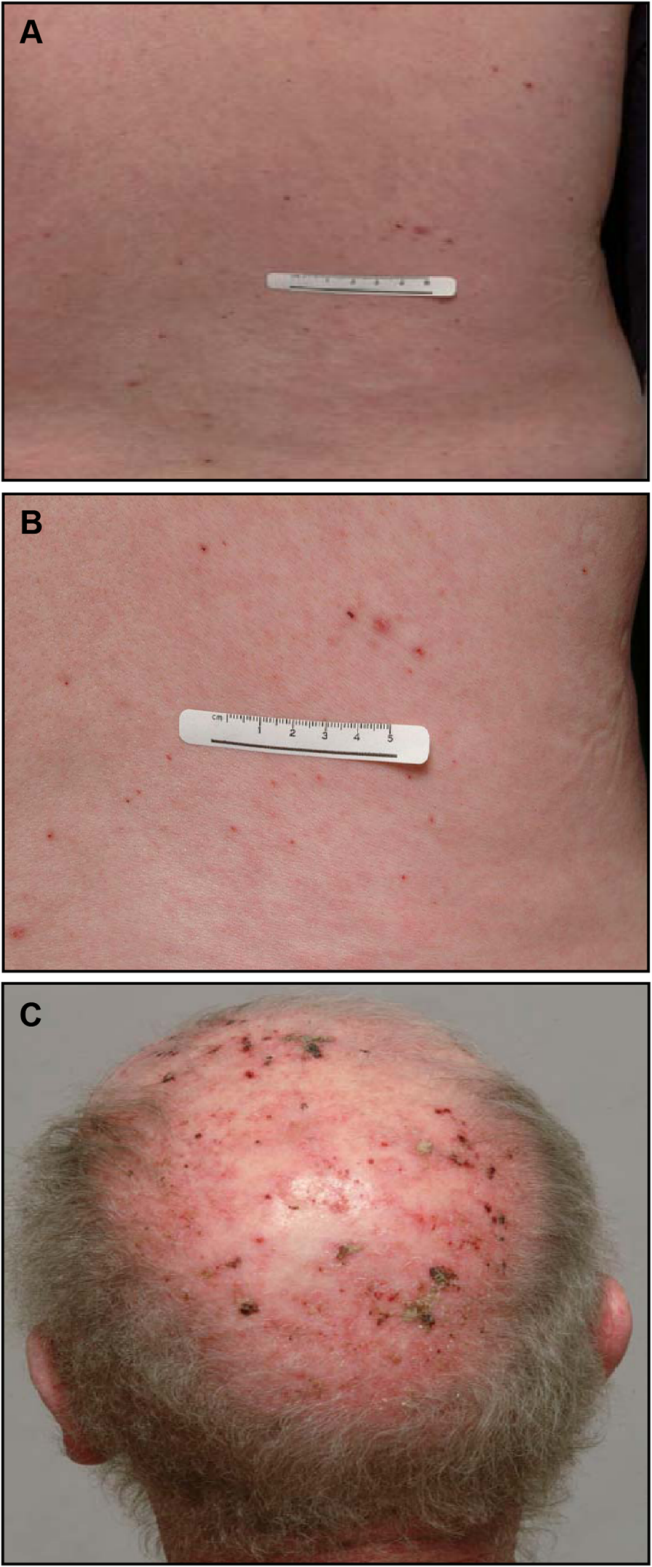
**Patterns of drug induced rash.** The typical rash associated with MK-2206 was a widespread, reversible, generalized, erythematous, maculopapular, non-acneiform rash **(A and B)**. In arm 3, some patients also demonstrated an acneiform rash affecting the head, face, neck, and trunk, in addition to the maculopapular MK-2206 related rash **(C)**.

In arm 1, preplanned dose escalation from 45 to 60 mg was achieved; DLTs were rash (1 of 5 at 45 mg and 1 of 4 at 60 mg; Figure 1A and 1B) and febrile neutropenia (2 of 4 at 60 mg). All episodes of rash were fully reversible within 7 to 14 days after dose interruption and appropriate treatment. The MTD of MK-2206 was established as 45 mg PO, when administered on the QOD dose schedule on days 1, 3, 5, and 7, with carboplatin AUC 6 and paclitaxel 200 mg/m^2^. In arm 2, when MK-2206 was administered at 45 mg days 1–7 QOD with docetaxel 75 mg/m^2^, 3 of 5 patients experienced a DLT of febrile neutropenia. In arm 3, DLTs were rash and stomatitis (2 of 8 at MK-2206 45 mg with erlotinib 100 mg; 1 of 3 at MK-2206 45 mg with erlotinib 150 mg). The rash seen in patients treated with MK-2206 and erlotinib was a combination of the acneiform pattern seen with erlotinib and the maculopapular pattern associated with MK-2206 (Figure 1C). Based on MK-2206 single-agent PK data and the high incidence of febrile neutropenia in arm 2 with docetaxel 75 mg/m^2^, a decision was made to reduce the dose of docetaxel to 60 mg/m^2^ and investigate QW and Q3W schedules.

### Q3W and QW dosing schedules

In arms 1 and 2, dose escalation through 3 dose levels administered Q3W (90, 135, and 200 mg) was achieved. In arm 1, DLTs were rash in 1 of 5 and 2 of 5 patients at 90 mg and 200 mg, respectively, and thrombocytopenia in 1 of 5 patients at 135 mg. Therefore, the MTD of Q3W MK-2206 dosing in combination with carboplatin and paclitaxel was 135 mg. In arm 2, the only DLT was tinnitus in 1 of 4 patients at MK-2206 200 mg with a reduced dose of docetaxel at 60 mg/m^2^. The combination was not to be investigated further, so this dose level was defined as the MAD; the MTD was not reached. In arm 3, 135 mg MK-2206 administered QW was tested with erlotinib 100 mg and 150 mg, with 1 DLT of rash observed in 5 patients at the higher dose level of erlotinib. As DLTs were low (17%), the MTD of MK-2206 in this combination was not reached. The MAD was defined as MK-2206 135 mg QW with erlotinib 150 mg.

### Safety and tolerability

Overall, the treatment combinations were well-tolerated, with a low incidence of grade 3 or 4 AEs. The most common AEs were similar across the arms and schedules—fatigue (68%), nausea (49%), rash (47%), diarrhea (44%), anorexia (44%), alopecia (40%), vomiting (36%), stomatitis (32%), and hyperglycemia (25%; 8% considered drug-related). Hyperglycemia was transient, grade 1/2, and largely associated with steroid premedication in arms 1 and 2. In arm 1, rash was observed more frequently with the days 1–7 QOD dose schedule (40%) compared with the Q3W schedule (25%). In arm 2, the frequency of febrile neutropenia decreased with dose reduction of docetaxel and change to Q3W dosing for MK-2206. There were no appreciable differences in the frequency of AEs between the QOD and QW schedules in arm 3. In arm 3 however, chronic anorexia, fatigue, rash and diarrhea, albeit at grade 1/2, were prominent. Table 4 summarizes treatment-related AEs observed by treatment arm and NCI-CTCAE grade.

**Table 4:** Treatment-related adverse events occurring in ≥20% of patients in any treatment arm with QOD and Q3W dosing schedules of MK-2206

**Adverse event, n (%)**	**Arm 1: carboplatin AUC 6;**					**Arm 2: docetaxel**				**Arm 3: erlotinib**			
	**Paclitaxel 200 mg/m**^**2**^					**75 mg/m**^**2**^	**60 mg/m**^**2**^			**100 mg and 150 mg**			
	**MK-2206**		**MK-2206**			**MK-2206**	**MK-2206**			**MK-2206**	**MK-2206**		
	**QOD**		**Q3W**			**QOD**^**a**^	**Q3W**^**b**^			**QOD**	**QW**		
	**45 mg (n = 6)**	**60 mg (n = 9)**	**90 mg (n = 5)**	**135 mg (n = 5)**	**200 mg (n = 6)**	**45 mg (n = 5)**	**90 mg (n = 3)**	**135 mg (n = 4)**	**200 mg (n = 4)**	**45 mg**^**c **^**(n = 9)**	**45 mg**^**d **^**(n = 4)**	**135 mg**^**c **^**(n = 6)**	**135 mg**^**d **^**(n = 6)**
Fatigue													
Grade 1/2	5 (83)	6 (66)	4 (80)	4 (80)	1 (17)	4 (80)	3 (100)	2 (50)	4 (100)	4 (44)	3 (75)	4 (66)	3 (50)
Grade 3	0	0	0	1 (20)	0	0	0	0	0	1 (11)	0	0	1 (17)
Grade 4	0	0	0	0	0	0	0	0	0	0	0	0	0
Nausea													
Grade 1/2	3 (50)	5 (55)	3 (60)	2 (40)	4 (66)	2 (40)	1 (33)	1 (25)	2 (50)	2 (22)	4 (100)	1 (17)	2 (33)
Grade 3	0	1 (11)	1 (20)	0	0	0	0	0	0	1 (11)	0	0	0
Grade 4	0	0	0	0	0	0	0	0	0	0	0	0	0
Rash													
Grade 1/2	3 (50)	1 (11)	0	1 (20)	1 (17)	4 (80)	2 (66)	0	1 (25)	3 (33)	1 (25)	4 (66)	5 (83)
Grade 3	1 (17)	1 (11)	1 (20)	0	1 (17)	0	0	0	0	2 (22)	2 (50)	0	0
Grade 4	0	0	0	0	0	0	0	0	0	0	0	0	0
Decreased appetite													
Grade 1/2	2 (33)	5 (55)	2 (40)	2 (40)	2 (33)	2 (40)	0	2 (50)	1 (25)	1 (11)	4 (100)	3 (50)	5 (83)
Grade 3	0	0	0	1 (20)	0	0	0	0	0	1 (11)	0	0	0
Grade 4	0	0	0	0	0	0	0	0	0	0	0	0	0
Diarrhea													
Grade 1/2	1 (17)	4 (44)	2 (40)	1 (20)	2 (33)	1 (20)	2 (66)	0	2 (50)	4 (44)	3 (75)	4 (66)	5 (83)
Grade 3	0	0	0	0	0	0	0	0	0	1 (11)	0	0	0
Grade 4	0	0	0	0	0	0	0	0	0	0	0	0	0
Alopecia													
Grade 1/2	4 (66)	6 (66)	5 (100)	4 (80)	2 (33)	3 (60)	1 (33)	1 (25)	2 (50)	0	0	1 (17)	0
Grade 3	0	0	0	0	0	0	0	0	0	0	0	1 (17)	0
Grade 4	0	0	0	0	0	0	0	0	0	0	0	0	0
Vomiting													
Grade 1/2	1 (17)	3 (33)	0	3 (60)	4 (66)	3 (60)	1 (33)	1 (25)	1 (25)	4 (44)	1 (25)	1 (17)	0
Grade 3	0	1 (11)	1 (20)	0	0	0	0	0	0	0	0	1 (17)	0
Grade 4	0	0	0	0	0	0	0	0	0	0	0	0	0
Anemia													
Grade 1/2	1 (17)	1 (11)	2 (40)	1 (20)	1 (17)	2 (40)	0	1 (25)	1 (25)	2 (22)	0	0	2 (33)
Grade 3	0	3 (33)	1 (20)	1 (20)	0	0	0	0	0	0	0	1 (17)	0
Grade 4	1 (17)	1 (11)	0	1 (20)	0	0	0	0	0	0	0	0	1 (17)
Stomatitis													
Grade 1/2	1 (17)	2 (22)	2 (40)	1 (20)	1 (17)	2 (40)	0	1 (25)	0	3 (33)	1 (25)	4 (66)	4 (66)
Grade 3	0	0	0	0	0	0	0	0	0	1 (11)	0	0	0
Grade 4	0	0	0	0	0	0	0	0	0	0	0	0	0
Neutropenia													
Grade 1/2	0	0	0	0	0	0	0	0	0	0	0	0	0
Grade 3	0	2 (22)	2 (40)	1 (20)	1 (17)	1 (20)	1 (33)	0	0	0	0	0	0
Grade 4	1 (17)	3 (33)	2 (40)	2 (40)	0	2 (40)	1 (33)	0	1 (25)	0	0	0	0
Leukopenia													
Grade 1/2	1 (17)	3 (33)	0	1 (20)	0	0	1 (33)	0	1 (25)	0	0	0	0
Grade 3	0	1 (11)	4 (80)	2 (40)	1 (17)	0	0	0	0	0	0	0	0
Grade 4	0	2 (22)	0	1 (20)	0	1 (20)	0	0	0	0	0	0	0
Thrombocytopen-ia													
Grade 1/2	0	4 (44)	1 (20)	1 (20)	1 (17)	2 (40)	1 (33)	0	0	0	0	0	1 (17)
Grade 3	1 (17)	1 (11)	1 (20)	0	0	0	0	0	0	0	0	0	0
Grade 4	0	0	0	2 (40)	0	0	0	0	0	0	0	0	0
Pruritus													
Grade 1/2	4 (66)	0	0	1 (20)	1 (17)	2 (40)	0	0	0	3 (33)	0	2 (33)	2 (33)
Grade 3	0	0	0	0	0	0	0	0	0	0	0	1 (17)	0
Grade 4	0	0	0	0	0	0	0	0	0	0	0	0	0

^a^MK-2206 QOD explored with docetaxel 75 mg/m^2^ only.

^b^MK-2206 Q3W explored with docetaxel 60 mg/m^2^ only.

^c^100 mg erlotinib.

^d^150 mg erlotinib.

### Pharmacokinetics

Pharmacokinetic data for MK-2206 were available from 70 patients: 30 from arm 1, 15 from arm 2, and 25 from arm 3. Across all treatment arms, AUC, maximum plasma concentration (C_max_), and time to maximum concentration (T_max_) values following the first MK-2206 dose were within ranges observed at the corresponding dose levels in the single-agent phase 1 study [18]. However, due to insufficient PK sampling, steady-state exposure in combination with either carboplatin and paclitaxel, docetaxel or erlotinib could not be assessed. The apparent t_1/2_ of MK-2206 in combination with carboplatin and erlotinib was consistent with that observed with monotherapy. Table 5 shows the key PK parameters for MK-2206 at 45-mg, 60-mg, 90-mg, and 135-mg dose levels in this combination therapy study after the first dose. The mean 48-hr plasma concentrations of MK-2206 at the 45-mg QOD (arm 3), 60-mg QOD (arm 1), and 135-mg QW (arm 3) dose levels were above 56.8 nM, at the last PK sampling time corresponding to 70% AKT inhibition in the single-agent phase 1 study [18].

**Table 5:** Pharmacokinetic parameters of MK-2206 after the first dose when administered in combination therapy

**Treatment arm**		**MK-2206 dosing schedule**	**Number of patients**	**AUC**_**0-48h**_**, nM•h**^**a**^	**C**_**max**_**, nM**^**a**^	**T**_**max**_**, h**^**b**^	**t**_**1/2**_**, h**^**c**^
1	Carboplatin AUC 6 Paclitaxel 200 mg/m^2^	45 mg QOD	6	1630 ± 496 (30.4)	57.7 ± 13.8 (23.9)	4.0 (4.0–6.0)	NA
		60 mg QOD	8	2700 ± 619 (23.0)	88.3 ± 24.2 (27.4)	8.0 (6.0–10.0)	NA
		90 mg Q3W	5	4130 ± 1520 (36.6)	144 ± 57.0 (39.6)	6.0 (4.0–10.0)	79.5 ± 17.3
		135 mg Q3W	6	7600 ± 1280 (15.3)	255 ± 50.9 (6.8)	8.0 (6.0–10.0)	73.0 ± 20.0
		200 mg Q3W	5	9800 ± 2550 (25.9)	458 ± 268 (58.5)	4.0 (4.0–10.0)	74.7 ± 13.4
2	Docetaxel 75 mg/m^2^	45 mg QOD	5	1320 ± 395 (30.0)	42.9 ± 13.3 (30.9)	6.0 (4.0–10.0)	NA
	Docetaxel 60 mg/m^2^	90 mg Q3W	3	3000 ± 1250 (41.7)	106 ± 42.5 (40.2)	4.0 (4.0–10.0)	105.4 ± 15.0
		135 mg Q3W	3	8090 ± 542 (6.7)	278 ± 35.5 (12.8)	6.0 (4.0–10.0)	106.1 ± 32.7
		200 mg Q3W	4	7690 ± 1550 (20.1)	287 ± 67.6 (23.5)	6.0 (4.0–6.0)	86.9 ± 11.5
3	Erlotinib 100 mg	45 mg QOD	9	1460 ± 417 (28.6)	48.8 ± 11.2 (23.0)	6.0 (4.0–10.0)	NA
		135 mg QW	6	6420 ± 2760 (42.9)	212 ± 75.9 (35.8)	6.0 (2.0–6.0)	60.6 ± 6.6
	Erlotinib 150 mg QD	45 mg QOD	4	2110 ± 637 (30.2)	65.6 ± 29.3 (44.6)	7.0 (4.0–24.0)	NA
		135 mg QW	6	6560 ± 2650 (40.2)	244 ± 84.2 (34.5)	4.0 (4.0–10.0)	50.2 ± 10.3

*Abbreviations*: *AUC*_*0-48h*_ area under the curve from 0 to 48 hours, *C*_*max*_ maximum concentration, *T*_*max*_ time to maximum concentration, *Q21* every 21 days, *NA* not available, *SD* standard deviation.

^a^Mean ± SD (coefficient of variation%).

^b^Median (range).

^c^Harmonic mean ± pseudo SD.

An analysis of docetaxel PK samples did not indicate a PK cause for the higher-than-expected frequency of febrile neutropenia DLTs in arm 2: the mean end-of-infusion concentration value was 3.01 μg/mL. This is within the range of mean values of 1.68 to 4.06 μg/mL reported in the literature [23-25]. Paclitaxel, erlotinib, and carboplatin plasma samples were not assayed. Paclitaxel is metabolized by CYP3A and CYP2C, while erlotinib is metabolized primarily by CYP3A and CYP1A. The major drug-metabolizing enzyme for docetaxel is CYP3A; thus, docetaxel is vulnerable to CYP3A-mediated drug-drug interactions, while carboplatin undergoes renal excretion and hepatic metabolism equally, reducing its potential to be a victim of CYP-mediated DDIs. MK-2206 is not a significant inhibitor or inducer of major CYP enzymes (IC50 >35 μM for CYP3A4, 2C9, and 2D6 inhibition, and has insignificant effect on CYP3A mRNA and activity at 0.1 to 10 μM); therefore, MK-2206 is not expected to perpetuate significant drug-drug interactions at the clinical doses. MK-2206 is susceptible as a victim to CYP3A-mediated drug-drug interaction as metabolism of MK-2206 to oxidative metabolites in human microsomes is mediated primarily by CYP3A. Exposure of MK-2206 after co-administration with erlotinib did not suggest a substantial PK interaction of MK-2206 as a victim, although attainment of a steady state of MK-2206 could not be confirmed at the last PK sampling. Steady-state exposure of MK-2206 in combination with either carboplatin and paclitaxel or docetaxel are not available based on the study design used.

### Antitumor activity and biomarker analysis

**Figure 2:**
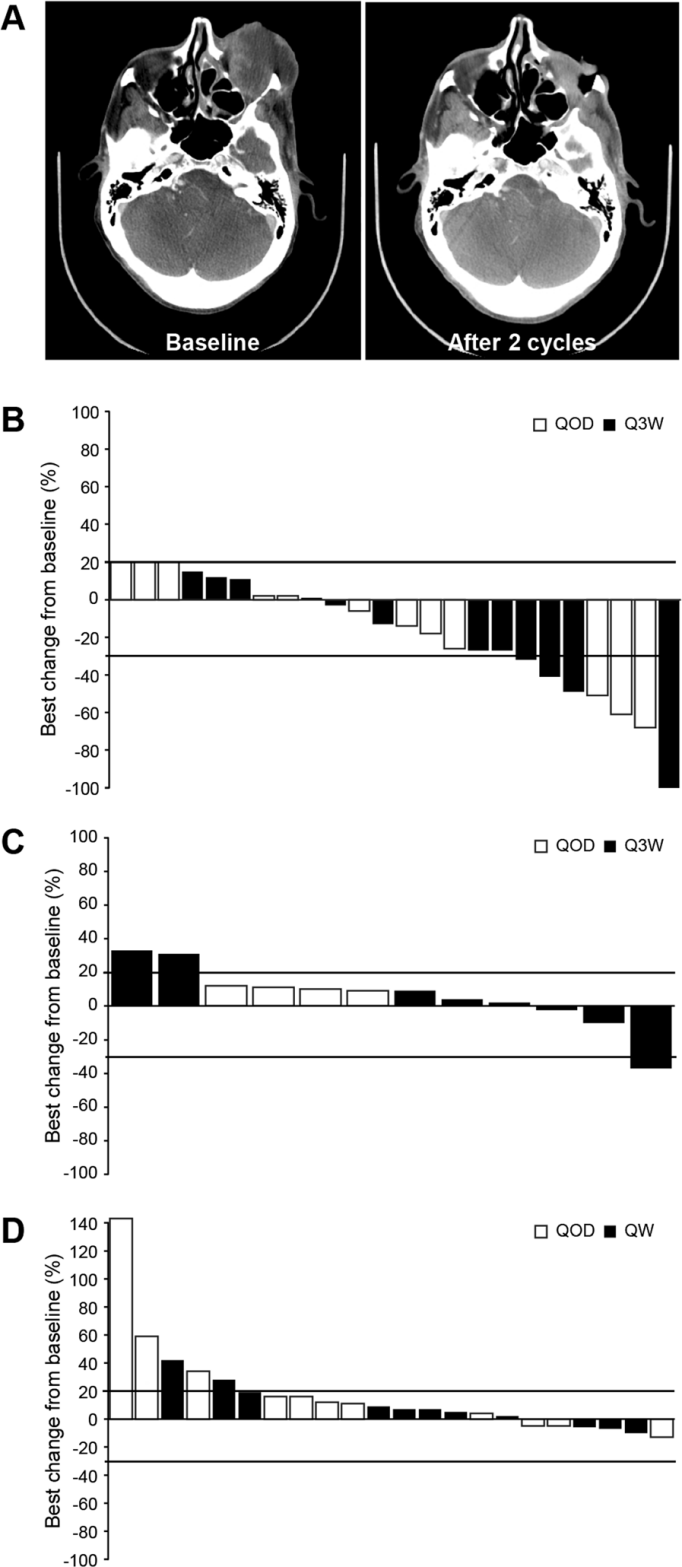
**Best radiological response (RECIST) associated with MK-2206 combinations.** CT scan slices demonstrating a CR in a male patient with SCC of the orbit **(A)**. Waterfall plots of the best responses seen in all evaluable patients in arm 1, carboplatin and paclitaxel **(B)**, arm 2, docetaxel **(C)**, and arm 3, erlotinib **(D)**. In arm 1, 6 patients treated in both schedules of MK-2206 with carboplatin and paclitaxel chemotherapy had PRs: with 4 of these being confirmed: melanoma (n = 1, 21-month duration—8 months while on study and 13 months after study discontinuation), neuroendocrine prostate (n = 1, 6-month duration), cervical (n = 1, 6-month duration), and endometrial (n = 1, 4-month duration); and 2 were unconfirmed: gastric (n = 1, 2-month duration) and SCC of the head and neck (n = 1, 2-month duration). In arm 2, a female patient with NSCLC who had progressed through pemetrexed-platinum and erlotinib achieved a PR with MK-2206 200 mg lasting 6 months. The patient withdrew from the study due to docetaxel-related toxicities, before documentation of progressive disease. No objective responses were observed in arm 3: the best response was SD lasting 7 and 6 months in a male patient with NSCLC and a patient with cervical cancer, respectively. Dashed lines indicate the threshold for PD (≥20% increase) and PR (≥30% decrease) based on the change in the sum of target lesions from baseline by Response Evaluation Criteria in Solid Tumors guidelines. CT = computed tomography; CR = complete response; SCC = squamous cell carcinoma; PR = particle response; NSCLC = non-small cell lung cancer; SD = stable disease; PD = progressive disease; QOD = alternate days; Q3W = every 3 weeks; QW = week.

Sixty patients were evaluable for response (arm 1, n = 24; arm 2, n = 13; arm 3, n = 23). In arm 1, a complete response was observed in 1 patient with squamous cell carcinoma (SCC) of the orbit who had progressed through previous cisplatin, cetuximab, and 5-fluorouracil therapy (Figure 2A). Four patients treated on both the days 1–7 QOD and Q3W schedules in arm 1 had confirmed partial responses (PR; melanoma, neuroendocrine prostate, endometrial, and cervical), and 2 patients had unconfirmed PRs (SCC of the head and neck, and gastric; Figure 2B). The patient with neuroendocrine prostate cancer had previously had a best response of stable disease (SD) with carboplatin and etoposide, while the patient with endometrial cancer had demonstrated SD with carboplatin and paclitaxel. The median duration of PR was 11 months (range 2–21 months). Six patients demonstrated SD lasting at least 6 months (median duration of SD was 7 months [range 1–13]). One patient with non-small cell lung cancer (NSCLC) treated Q3W with MK-2206 and docetaxel demonstrated a confirmed PR; this patient had received 2 prior lines of platinum-based chemotherapy and erlotinib. Figures 2C and 2D illustrate the overall best responses in evaluable patients in arms 2 and 3, respectively. Biomarker analysis was conducted on 68 patients, of whom 7 tested positive for mutations. Four patients carried a *PIK3CA* mutation (exon 20) and 3 carried a *KRAS* (exon 12) mutation. Patients harboring *PIK3CA* mutations demonstrated SD ranging from 1.7 to 5.8 months, whereas patients with *KRAS* mutations had SD ranging from 1.4 to 3.0 months.

## Discussion

Our study demonstrates that MK-2206 in combination with standard cytotoxic chemotherapies can be safely administered to patients with advanced solid tumors, at doses demonstrating antitumor activity. The combinations were tolerable, with the main DLTs being rash and febrile neutropenia. The most common AEs were fatigue, nausea, rash, diarrhea, and anorexia, with no apparent exacerbation of toxicities associated with standard agents. Of note, drug-related hyperglycemia—an expected effect of AKT inhibition—was evident in <10% of patients, a rate similar to the single-agent phase 1 study [18].

At the 45-mg dose level in all schedules, mean C_trough_ for MK-2206 was >56.8 nM, corresponding with 70% AKT inhibition in the single-agent study [18]. The MTD and recommended schedule of MK-2206 in combination with carboplatin AUC 6 and paclitaxel 200 mg/m^2^ were defined as 135 mg Q3W. With docetaxel, the MTD of MK-2206 was not reached, but the MAD of MK-2206 was defined as 200 mg Q3W with docetaxel 60 mg/m^2^. The dose escalation of this combination was not further investigated due to a high neutropenia rate when docetaxel was administered at 75 mg/m^2^ and the lack of clinical activity; this 60% rate of febrile neutropenia may be a reflection of the small patient numbers. However, it was felt that these 2 factors would likely limit the future use of this combination. The MK-2206 plus erlotinib combination reached the MAD of 135 mg QW and 150 mg daily, respectively. Based on emerging data from the monotherapy phase 1 study where MK-2206 MTD is limited to 200 mg QW, as well as the overlapping skin toxicity profiles of these two agents, it was decided that the risk of further dose escalation outweighed the potential benefit and therefore the MAD is the recommended phase 2 dose (RP2D).

The anticancer activity of the combinations, particularly MK-2206/carboplatin and paclitaxel, was notable, with durable responses seen in some patients previously exposed to platinum and taxane compounds. There was no correlation between responses or prolonged SD with mutations activating the PI3K pathway, although 1 metastatic breast cancer patient harboring a *PIK3CA* mutation (exon 20), previously treated with 3 lines of chemotherapy and 1 line of hormonal therapy with progressive disease, demonstrated SD for 4 months. Phase 1 studies of MK-2206 with paclitaxel in breast cancer and with paclitaxel and trastuzumab in patients overexpressing human epidermal growth factor receptor-2 are underway.

Preclinical data in NSCLC cell lines suggest a strong rationale for the combination of MK-2206 and erlotinib [19,26]. In our study, most patients with NSCLC (n = 5) were treated on the less well-tolerated and possibly suboptimal QOD schedule. Using the QW schedule, only 1 patient with NSCLC, who achieved a best response of SD lasting 7 months, was included. The antitumor activity of this combination is likely to be best determined in selected patients with molecularly characterized tumors. The combination is currently being investigated in an open-label, phase 2 trial of patients with advanced NSCLC. A phase 1 study is also investigating the combination of MK-2206 with gefitinib in patients who progressed on prior treatment with an epidermal growth factor receptor (erlotinib or gefitinib) inhibitor. Previous results suggest that tumors with *KRAS* mutations are more effectively inhibited with a combination of an AKT and a MEK inhibitor [27,28]. These combinations are being further explored in the Biomarker Integrated Targeted Therapy Program (BATTLE-2), where recruitment into different arms is based on the molecular status of the patient’s tumor [29].

Based on the early evidence of clinical activity, larger phase 2 randomized studies are underway in various tumor types to test whether the addition of MK-2206 to standard treatment enhances antitumor effects. One such study is assessing the combination with the anti-androgen bicalutamide, where patients with prostate cancer are randomized to receive bicalutamide with or without MK-2206. Dysregulation of the PI3K pathway is one of the most frequent mechanisms of resistance to conventional anti-androgen therapy [30], highlighting a need for effective agents that could inhibit cell signalling via this pathway. Another study is investigating the efficacy of the addition of MK-2206 to anastrazole or fulvestrant, and comparing these combinations to either agent alone in women with metastatic breast cancer.

In conclusion, our study shows that MK-2206, using a QOD, QW, or Q3W dosing schedule in combination with carboplatin and paclitaxel, docetaxel, or erlotinib, was well-tolerated at doses that inhibit AKT signaling. Phase 2 programs are underway to further investigate the combination of MK-2206 with carboplatin and paclitaxel or erlotinib, which along with other randomized phase 2 studies should provide a broad clinical profile of MK-2206 in combination with other standard cytotoxic or targeted treatment options.

## Abbreviations

AEs: Adverse events; ATP: Adenosine triphosphate; BATTLE-2: Biomarker integrated targeted therapy program; CT: Computed tomography; DLT: Dose-limiting toxicities; MAD: Maximum administered dose; MTD: Maximum tolerated dose; mTOR: mammalian target of rapamycin; NCI-CTCAE: National cancer institute common terminology criteria for adverse events; NSCLC: Non-small cell lung cancer; PD: Pharmacodynamics; PI3K: Phosphatidylinositide 3-kinase; PK: Pharmacokinetics; PO: Orally; QOD: Alternate day; QW: Weekly; Q3W: Once every 3 weeks; RP2D: Recommended phase 2 dose; SD: Stable disease.

## Competing interests

The following authors declare financial interest in the work presented in the submitted manuscript: LRM, DMS, LLS, AP, and KPP have received research funding from Merck & Co., Inc. LY and ET are employees of and own stock in Merck. AMZ is an employee of Merck & Co., Inc. AWT has acted as a consultant or advisor for AbbVie, Aragon, Arqule, Astellas, Astex (formerly Supergen), Bayer HealthCare, Bind Biosciences, BioMed Valley, Bristol-Myer Squibb, Celator, Curis, Cytomx, Dicerna, EMD Serono, Inc., Endo, Five Prime Therapeutics Inc., Genentech, Lilly, Merck & Co., Inc., Mercus, Nanobiotx, Nektar, Neumedicines, Novartis, OncoMed, Pfizer, Pierre Fabre, Prism Pharma Co., Ltd, ProNai, Sanofi, Specialized Medical Services-Oncology BV, Sympogen, Theraclone, Vaccinex, and Zyngenia. JV-P, PAC, EC, AB, JSdB, and SM have no conflicts of interest to disclose.

## Authors’ contributions

LRM collected and assembled data and interpreted and analyzed the data, and participated in writing the manuscript. JV-P participated in writing of the manuscript. AMZ assisted with conception and design of the study, collected and assembled data, and conducted data analysis and interpretation; participated in writing the manuscript; and provided administrative support and provided patients or study materials. DMS conducted data analysis and interpretation and participated in writing the manuscript. PAC and EC collected or assembled data and participated in writing the manuscript. AB collected and assembled data and participated in writing the manuscript. ET interpreted and analyzed the data and participated in writing the manuscript. LLS collected and assembled the data, provided patients or study materials and participated in writing the manuscript. AP collected and assembled the data and participated in writing the manuscript. KPP collected and assembled the data, provided patients or study materials and participated in writing the manuscript. JdB assisted with conception and design of the study and collection and assembly of data and provided study material or patients. AWT assisted with conception and design of the study, collection and assembly of data, and did data analysis and interpretation; participated in writing of the manuscript; and provided study material or patients. SM assisted with conception and design of the study, did data analysis and interpretation, and participated in writing the manuscript. All authors approved the final version of the manuscript. All authors read and approved the final manuscript.
